# Evaluation of phthalate migration potential in vacuum-packed

**DOI:** 10.1038/s41598-024-54730-5

**Published:** 2024-04-04

**Authors:** Gonca Alak, Mine Köktürk, Muhammed Atamanalp

**Affiliations:** 1 Department of Seafood Processing Technology, Faculty of Fisheries, Ataturk University, TR-25030 Erzurum, Turkey; 2 Department of Organic Agriculture Management, Faculty of Applied Science, Igdir University, TR- 76000 Igdir, Turkey; 3 Department of Aquaculture, Faculty of Fisheries, Ataturk University, TR-25030 Erzurum, Turkey

**Keywords:** Phthalate ester, Whiting fish, Red mullet, Vacuum packaging, µ-Raman, Risk factors, Materials science

## Abstract

In recent years, the presence and migration of PAEs in packaging materials and consumer products has become a serious concern. Based on this concern, the aim of our study is to determine the possible migration potential and speed of PAEs in benthic fish stored in vacuum packaging, as well as to monitor the storage time and type as well as polyethylene (PE) polymer detection.As a result of the analysis performed by µ-Raman spectroscopy, 1 microplastic (MP) of 6 µm in size was determined on the 30th day of storage in whiting fish muscle and the polymer type was found to be Polyethylene (PE) (low density polyethylene: LDPE). Depending on the storage time of the packaging used in the vacuum packaging process, it has been determined that its chemical composition is affected by temperature and different types of polymers are formed. 10 types of PAEs were identified in the packaging material and stored flesh fish: DIBP, DBP, DPENP, DHEXP, BBP, DEHP, DCHP, DNOP, DINP and DDP. While the most dominant PAEs in the packaging material were determined as DEHP, the most dominant PAEs in fish meat were recorded as BBP and the lowest as DMP. The findings provide a motivating model for monitoring the presence and migration of PAEs in foods, while filling an important gap in maintaining a safe food chain.

## Introduction

With the integration of disposable materials into our lives in line with today’s social trends, technological changes and developments in the field of engineering, a rapid plastic use potential has emerged in the food sector. This contributes significantly to the global plastic pollution crisis, especially with the widespread use of plastic packaging for the storage, transportation and proper preparation or serving of foodstuffs. Innovative packaging techniques with advanced functionality are needed to meet these increasing demands.

In this sense, the global production of plastics made from various organic polymers (polyethylene (PE), polypropylene (PP), nylon, etc.) is gradually increasing^1,2^. Some plastics such as expanded polystyrene (EPS) or extruded polystyrene (XPS) are widely used in food packaging because they provide a good barrier to oxygen, water vapor and microorganisms, and preserve food at the desired temperature^3^. In plastic packaging, phthalates (PAEs) are organic lipophilic compounds commonly used as plasticizers and for the purpose of giving transparency to the product. These chemicals show particularly high migration to oil-based matrices due to reasons such as short half-lives, not being bound to polymers by covalent bonds and having a high tendency to leave the polymer in which they are located^4,5^. In addition to being used to soften plastic, it is known that they are preferred in many cosmetic products, toys, detergents and personal care products, as well as for the fragrance and coloring of products we frequently use in our daily life. Due to their widespread use and migration tendencies of phthalates (PAEs), they adversely affect the ecological environment as they are easily mixed with lakes, rivers and seas through accumulation in natural waters^6^. Although the use of PAEs in food contact materials is restricted by legislation, since it is a fat-soluble substance, it can be found especially in fatty foods, food products stored in oil and sauce, and can reach dimensions that may pose a health risk in long periods of time. The amount of PAE, whose migration increases with light and heat, should be monitored in foods.

Information on plastic migration in packaged seafood is limited to only a few studies^7–9^. In this study, prepared to fill this gap, the presence and formation of PAEs in packaged, unpackaged fish meat, and packaged stored flesh fish, as well as the migration potential and rate depending on the storage period were investigated. Secondly, with a deterministic approach, in addition to monitoring the efficiency and speed of possible migration of oily and lean fish, it is aimed to detect packaging-origin polyethylene (PE) polymer.

## Materıal and methods

### Fish supply, vacuum packaging and storage operations

#### Fish material

In the study, benthic fish [oily (red mullet, *Mullus barbatus*) and lean (whiting fish, *Merlangius merlangus euxinus*)] obtained from a commercial company were used. The experiment was designed by randomly distributing 25 fish (average weight 27 g) to each package (3 replications).

#### Vacuum packaging

The packaging process was done in bags with a size of 15 × 25 cm and low gas permeability (at 23 °C, oxygen permeability: 5 cm^3^/m^2^/day atm, Nitrogen permeability: 1 cm^3^/m^2^/day atm, carbon dioxide permeability: 23 cm^3^/m^2^/day atm, water vapor permeability of 15 g/m^2^/day atm 38 °C). A total of 36 vacuum bags were used.

#### Storage

The vacuum packed flesh fish were stored for 3 months at the most commonly used commercial temperature (−18 °C)^10^. 36 vacuumed flesh fish were stored for 3 periods and the following analyzes were applied in the specified periods (0, 1, and 3 months)^11^.

### Sample preparation for MP isolation

For muscle tissues of processed fish samples (~ 3 g, 3 replicates from each group) procedures used in previous studies were used^12,13^. Tissue samples were incubated in a glass beaker with 10% KOH solution (3:1 solution: tissue) for 48 h at 60 °C until the organic matter was dissolved. After the digestion period, the residue was filtered through glass fiber filters (Whatman GF/C) using a vacuum pump. The filters were placed in a glass petri dish and incubated at 60 °C for 24 h.

The dried filters were examined under a stereomicroscope at 40 × magnification, with MPs characterized by color (black, blue, grey/white, red/pink, green and yellow) and shape (fiber, fragment and film). MP dimensions in the pictures were recorded under the microscope and measured using the ImageJ software program. The MP amount was calculated in terms of microplastic/g in muscle.

### a. Analysis of MPs by μ-Raman spectroscopy

μ-Raman spectroscopy (WITech alpha 300R) was used in Atatürk University DAYTAM Laboratory (Erzurum, TURKEY) to identify the chemical composition of the MPs on the filters isolated from the flesh fish and to confirm the identity of the particles. Detected MPs were morphologically characterized with × 50 objective (Olympus MPLAN, 50X, NA/0.55 LWD). It was then analyzed by Raman Microspectroscopy directly on the filter (spectral range 160–1800 cm^−1^, 785 nm laser).

The Open Specy Library (https://openanalysis.org/openspecy/, accessed date, June 2023) was used for the MP identification during µ-Raman spectroscopy, and they correlated more than 70% with reference polymers in the database^14,15^. In addition, the collected Raman spectra were compared with those reported in the SLoPP and SLoPP-E Raman Spectral Libraries of Microplastic spectral library. Scientific published data with a minimum library match of 70% is considered sufficient for verification^16^. Of the filters examined (*n* = 3, 18), only 2 filters contained microparticles and were subjected to spectroscopic verification.

### b. Analysis of package material by FTIR spectroscopy

Fourier transform infrared spectroscopy (FTIR) (Agilent Cary 630 FTIR Spectrometer, Agilent, Santa Clara, CA, ABD), which measures the spectral signal change of polymers in the wavelength range of 4000 to 650 cm^−1^, was used to determine the change in the chemical content of the material in fish packages (before packaging and after storage). The MPs’ polymer types are taken from the Agilent MicroLab FTIR software library. Spectra of the samples were compared with library data and samples with spectral similarity above 80% were accepted.

### c. Quality control and contamination prevention

All materials, equipment and open surfaces of the laboratory were wiped with pure water and ethanol to prevent contamination. All materials were stored in aluminum foil when not in use. In the course of the experiment, cotton laboratory coats were worn. After the laboratory was cleaned, the windows and ventilation of the laboratory were kept closed to prevent contamination from outside air. The samples were sealed as much as possible and processed as soon as possible. The procedural gaps (100 ml, 4 repetitions) of the KOH solution used in the study were filtered on Whatman filter paper and observed under a microscope. No microplastics were detected in the cavities.

All quality assurance and safety protocols prescribed in previous studies were followed while processing samples for microplastic analysis^17,18^. In addition, 20 laboratory blinds were kept and corrected from the final values in the study to control µ-Raman and FTIR airborne contamination in the laboratory during particle characterization.

### Periodic detection of the phthalate presence in stored vacuum flesh fish

Phthalate content was monitored once in the untreated flesh fish and vacuum bags however, in the stored flesh fish’ vacuum package were done periodically.

### Chemicals

Tetrahydrofuran (THF) CAS No:109-99-9, n-Hexane CAS No: 110-54-3, Cyclohexane CAS No: 110-82-7, Diisononyl phthalate (DINP) CAS 28553-12-0, Di-n-octyl phthalate (DNOP) CAS 117-84-0, Di-2-ethylhexyl phthalate (DEHP) CAS: 117-81-7, Diisodecyl phthalate (DIDP) CAS: 26761-40-0, Benzyl butyl phthalate (BBP) CAS: 85-68-7, Dibutyl phthalate (DBP) CAS: 84-74-2, Benzyl Benzoate (BB) (Internal Standart) CAS: 120-51-4, Diisobutyl phthalate (DIBP) CAS: 84-69-5, Dipentyl phthalate (DPENP) CAS: 131-18-0, Dihexyl phthalate (DHEXP) CAS: 84-75-3, Dicyclohexyl phthalate (DCHP) CAS: 84-61-7, and Acetonitrile (ACN) CAS: 75-05-08 were purchased from Merck. The purity of all chemicals used is over 97%.

### Material conditioning to prevent environmental pollution

No plastic materials were used throughout the experiments to avoid phthalate cross-contamination. All tools and glassware were heated in an ash oven at 350 °C for 72 h. Glassware was washed serially with dichloromethane and acetone. At the end of the phthalate determination studies, a reagent blank (blank) samplewas run with the studied samples to see the possibility of contamination from the materials, equipment or other chemicals used. No sample material was used when creating the blank.

### a. Samples preparation, extraction, and phthalate analysis

The procedure for phthalate extraction was adapted from the literature^19,20^. Typically, a 500 mg tissue sample was homogenized. The extraction of phthalates was performed by adding 10 mL of acetonitrile to each homogenized sample (500 mg) in a glass vial and by sonication of the resulting mixture for 20 min at room temperature. The extraction mixture was then centrifuged for 15 min at 3500 rpm. A portion of the extract supernatant (5 mL) was added to a volumetric flask^21^.

For each 5 mL of Acetonitrile, 10 mL of n-Hexane was added, shaken gently and the n-Hexane solvent was allowed to mix completely with the solution. It was left for at least 5 min for the polymer structure to collapse. 1 ml of the clear solution was taken and 10 µl of the Internal Standard Solution (100 mg/L) was added and the GC–MS vials were closed. Helium was used as the carrier gas at a flow rate of 1.0 mL min^−1^. In the analysis, the oven temperature program was set as follows: Held for 1 min at an initial temperature of 150 °C, held for 30 °C/min, from 150 to 280 °C, it was then increased from 280 to 310 °C at 15 °C/min and solvent delay time, 3 min The readings were done with injector temperature (at 290 °C) and the injections were made in splitless mode (20:1).

### Validation of the method

In our study, the “CPSC-CH-C1001-09.4 Standard Operating Procedure for Determination of Phthalates” analysis method was used. Specificity, Linearity, Recovery, Reproducibility, Reproducibility, Repeatability, measurement uncertainty, LOD and LOQ parameters were selected for validation of the analysis method. The analyses process was evaluated and validated according to the protocol described by AOAC International^22,23^.

For the evaluation of the selectivity parameter, the interferences due to the matrix and the chemicals used were evaluated with the data obtained by measuring the blank samples. Blank measurements did not yield any significant measurement values. This study proved that the Blank sample does not interfere with the analyte. The characteristic ions (m/z) of the phthalates were selected to look for matrix interferences at the retention times described.

**Figure 1:**
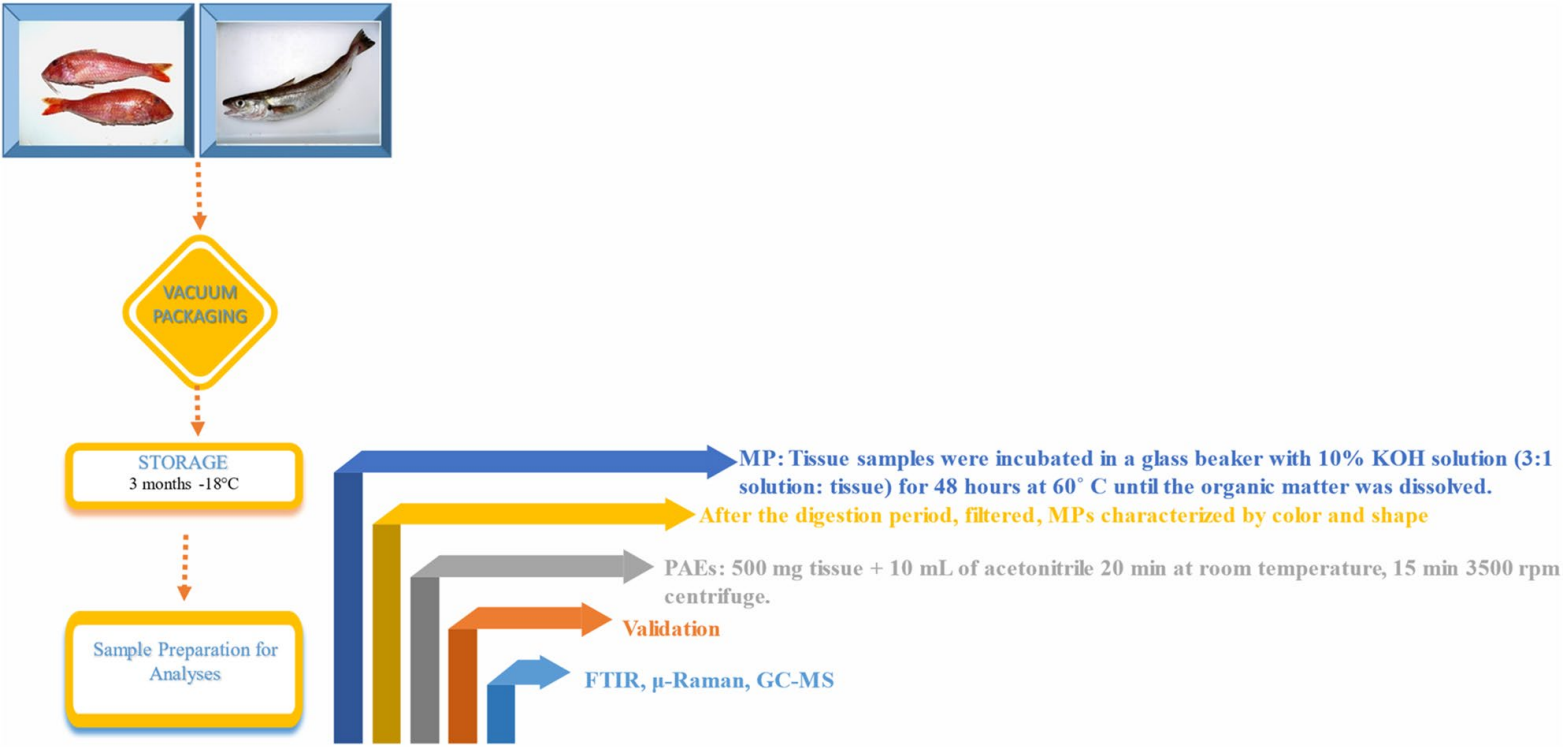
Samples preparation, extraction, and MP-PAEs analysis.

The following retention times (min) were recorded: DIBP: 4.91; DBP: 5.25; DPENP: 5.88; DHEXP: 6.53; BBP: 6.66; DEHP: 7.18; DCHP: 7.33, DNOP: 7.58; DINP: 7.80; DIDP: 8.51. This method selected two product ions and one precursor ion for each phthalate compound after injecting the samples into the GC–MS/MS. All analyzes procedure was showen Fig. 1.

Linearity Calibration solutions were read with 7 points and 3 parallels for each active substance. Linearity studies were considered appropriate because the correlation constant (R2) was greater than 0.995 and visually produced a linear graph. The uncertainty from linearity was calculated for each element using this graph and calculations.

Reproducible studies were performed at different levels for the recovery parameter. The evaluation of the recovery study was made according to the "AOAC Manual For The Peer Verified Methods Program Analyte—Recovery Table" table below.

In the validation studies, for the reproducibility parameter, studies were performed at the levels specified below. In-laboratory reproducibility study was performed by different employees, using the same device, on different days. F Test is used for the evaluation of the reproducibility study. The evaluation of the statistical test is performed according to the following condition Ftest > 1 and F_test_ < F _critical_.

Repeatability studies were carried out by each analyst using 6 test samples and parallel measurements. These studies were carried out by each analyst at different times, within the shortest period of time, under the same conditions and using the same instrument as described above. The results obtained according to the condition that the difference of the analysis results obtained from each test sample and parallel sample (× 1- × 2) is less than or equal to the repeatability limit value (r) are analyzed (x_1_-x_2_) ≤ r.

Parameters such as uncertainty from standard preparation, uncertainty from instruments and volumetric materials, linearity uncertainty, repeatability uncertainty, recovery uncertainty, reproducibility uncertainty were included in the measurement uncertainty calculation performed on the data obtained during validation studies.

With the uncertainty values obtained from all these parameters, the combined relative standard uncertainty was calculated and expanded according to the 95% confidence interval (k = 2) and the following values were obtained.

For the limit of detection (LOD) and limit of determination (LOQ) studies, 10 runs were performed for each sample at the lowest point of the linearity range. Validation studies were also performed for the values obtained. As a result of the studies, the LOD and LOQ, repeatability and measurement uncertainty values obtained for this analysis are given.

The values obtained in all validation studies were evaluated according to the validation parameters conditions mentioned above and the results were found to be appropriate.

### Statistical analysis

Two-way ANOVA was applied to statistically compare the results obtained from phthalate analyses, and Bonferroni's multiple comparison test was applied to make pairwise comparisons on individual groups (separately for time and species). The data were given as mean ± SD and statistical analyzes were made with GraphPad Prism 8 program. Differences were considered statistically significant at ****p < 0.0001, ***p < 0.001, **p < 0.01, and *p < 0.05.

## Results and discussion

**Figure 2:**
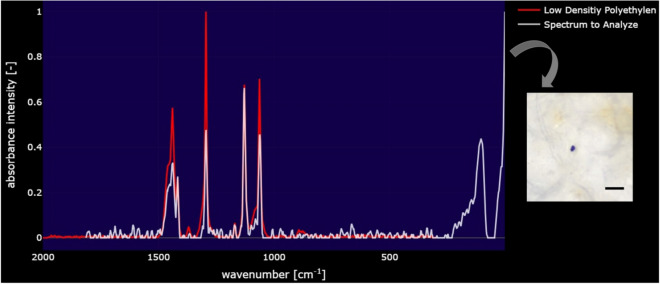
μ-Raman spectra, Low Density Polyethylene: LDPE. Scale bar: 100 µm.

The results of the researches that studied the negative effects of PAEs on the marine ecosystem organisms showed that these chemicals can be extremely dangerous in terms of their effects^24,25^. As these findings create a curiosity in processed seafood, the current research is a first for the migration of PAEs that will occur as a result of vacuum packaging of different fish species at the same temperature. The obtained findings revealed important data about the formation and migration of PAEs in benthic marine fish during the storage period. In this direction, as a result of the analysis made in µ-Raman spectroscopy, microplastic was determined in only one of them (Fig. 1). Microplastics were determined as a blue colored fragment of 6 µm in the fillet of *Merlangius merlangus euxinus* (−18 °C) in 30^th^ day samples. Characterization of 1 isolated microplastic by µ-Raman revealed that the polymer type was Polyethylene (PE) (low density polyethylene: LDPE) (Fig. 2). There have been very few studies on PAEs migration in food, and those that have focused beverages (water and coffee) with plastics. In a study conducted in this direction, PAEs homologs migrating into hot drinks as a result of the use of plastic cups were investigated and micron-sized (25,000) and submicron-sized (10.2 billion) microplastic migration was detected in a 15-min time period. In a study conducted in this direction, the homologs of PAEs that migrate to hot beverages as a result of the plastic cup use were studied and MP migration in micron size (25,000 units) and submicron size (10.2 billion) was determined in a 15-min time period^26^. When the results of our study are examined, it is seen that while the number of transitions (only 1) is not as high as in the study of Sewandi^26^, the determined microplastic size is consistent with it. It is quite clear that there are differences due to the type of polymer and food used here, as well as the temperature applied. The fragment size we determined in the flesh fish is compatible with Mason et al.^27^, and the researcher reported that MPs smaller than 100 µm can pass into food from processes such as packaging and/or bottling.

**Figure 3:**
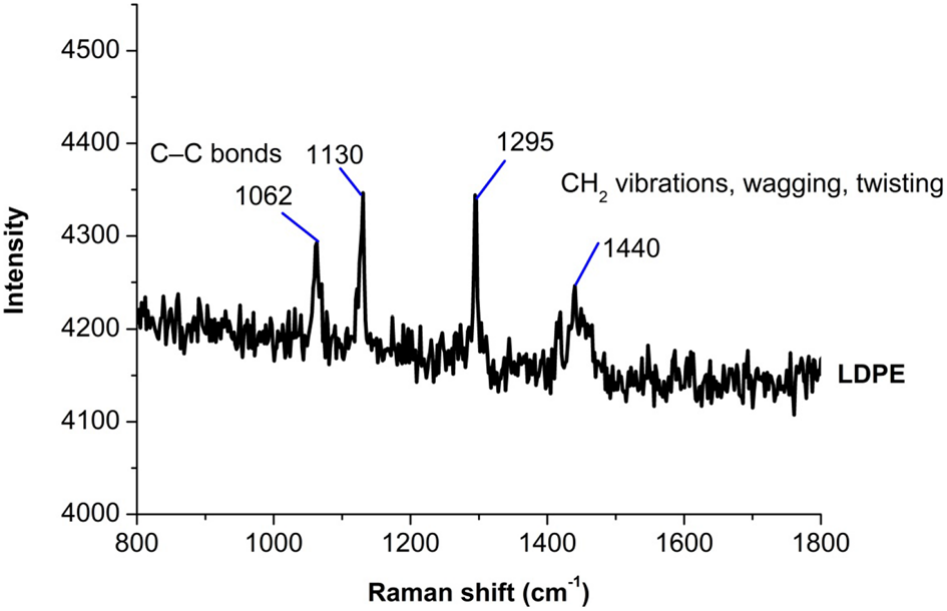
Raman spectra representing the Low Density Polyethylene MP species.

The main Raman shifts of PE are 1062, 1129, 1295, and 1440 cm^−1^^28^. These bands were determined as stretching vibrations of C–C bonds (1062 and 1129 cm^−1^) and bending of CH_2_ groups (1295 and 1440 cm^−1^)^29,30^. HDPE and LDPE are very similar, but the difference is the reported degrees of crystallinity in the C–H stretching region^31^. Compared to DBP, other phthalates such as DEHP, DBP, DIDP and DINP have similar core structures and longer—branched side chains. Branching in the carbon chain reduces the diffusion coefficient in polymers^32^. In our study, it was determined that the raman peak spectra of the microplastics we isolated from fish tissues were 1062, 1130, 1295, and 1440 cm^-1^, similar to the studies above (Fig. 3). Environmental conditions are effective in the migration of phthalates, and it has been revealed by the Arrhenius equation that there is more migration of these chemicals, especially with temperature increases. The small molecules of these chemicals, the free energy potential they have to increase migration from the polymer, increases with temperature, facilitating the breakdown of polymer-molecule interaction forces^32^. High temperatures cause swelling of the polymeric material, which causes the release of toxic substances in the package and their migration to the food, as it facilitates mass transfer^33,34^. However, this transfer may also occur by evaporation and mostly originating from the outer surface of the package during packaging compositions, stacking and storage of the package, and then pass into the food via the gas phase. In addition, these components and fibers may pass into food with recycled packaging materials^35^ and thus become part of the human diet unknowingly^36^.

**Figure 4:**
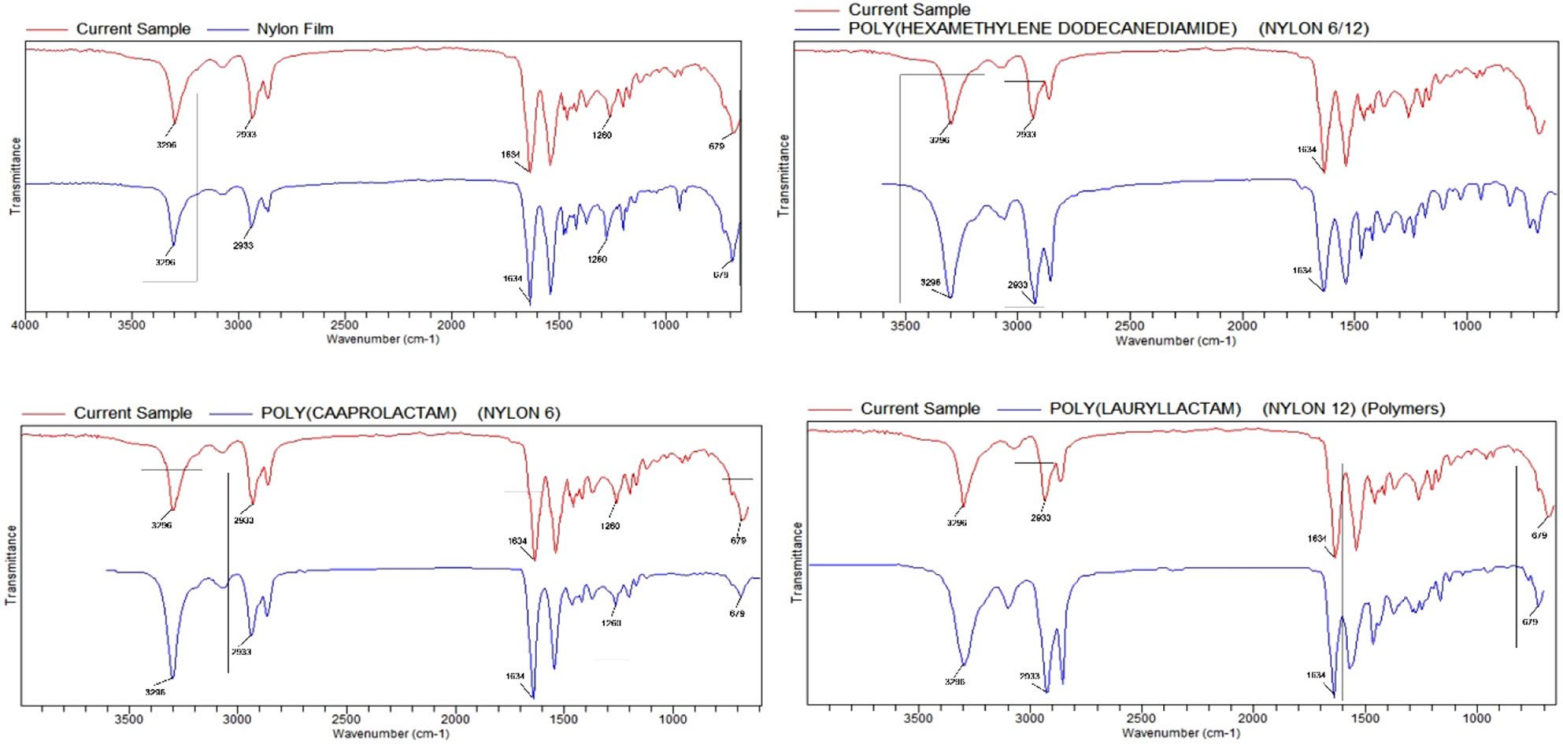
FTIR spectra of the chemical component of the package material before packaging (red line: package material, blue line: FTIR polymer reference).

**Figure 5:**
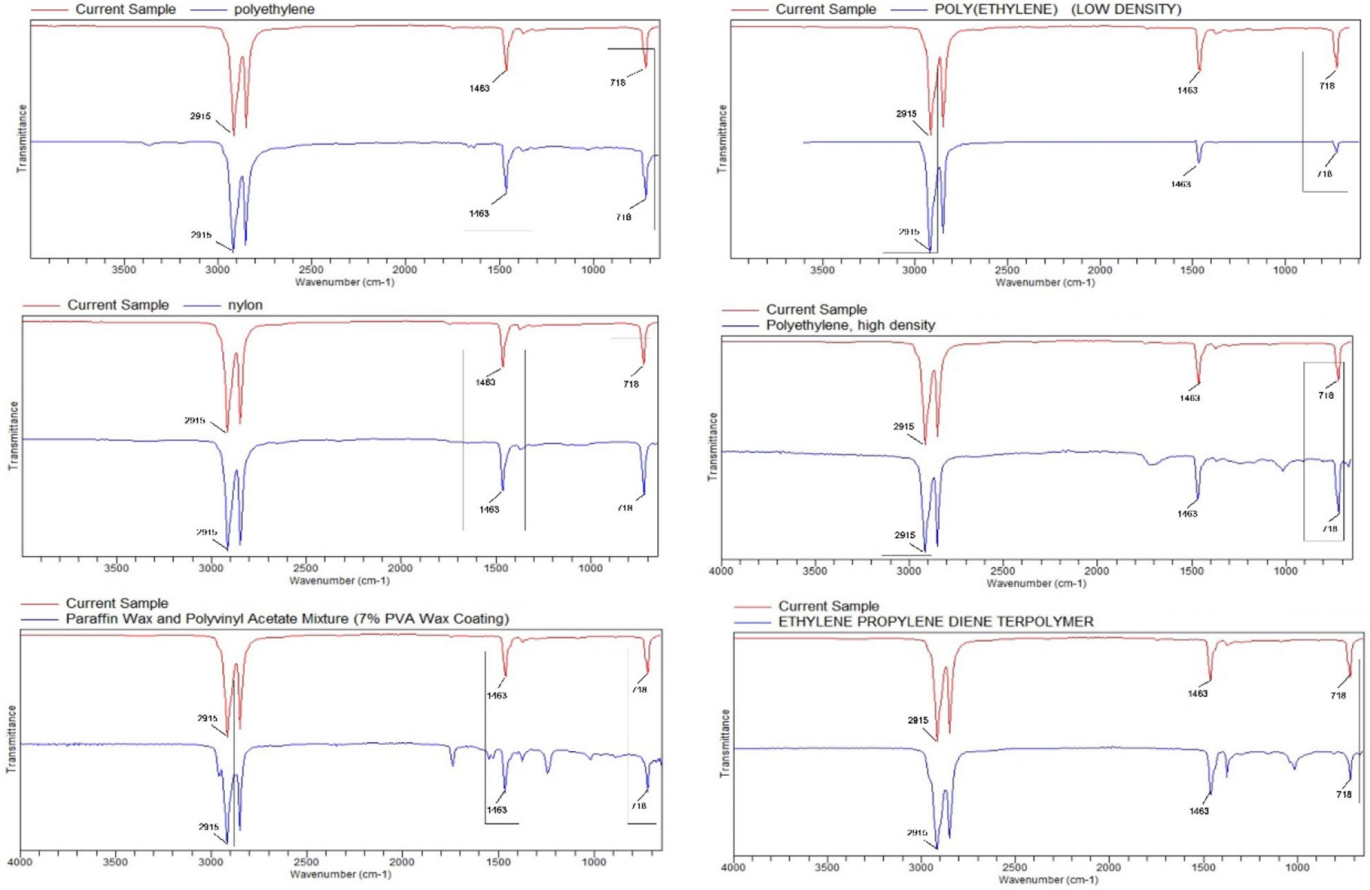
FTIR spectra of chemical components of processed package material (red line: package material, blue line: FTIR polymer referance).

The use of packages / containers made of polymer types [polyethylene (PE), polystyrene (PS), polypropylene (PP), and polyethylene terephthalate (PET)] in the food industry is a significant threat to the MP migrations. It is known that especially film food packages contain low-density polyethylene polymer type plastics^37^. Polyethylene (PE) is widely used in reusable bags and food packaging^38^. It is known that the daily use of polystyrene (PS) is in food packaging, plates, disposable cups, spoons^39^. In our study, it was determined that the vacuum packaging process and the subsequent storage time affected the chemical composition of the package material, and more different polymer types were detected in the processed package material (Figs. 4 and 5).

The higher temperature causes the polymeric material to swell, which facilitates both diffusion and mass transfer of some toxic substances in the polymeric material from the polymer to the food^33,34^.

In a study, it was found that the application of temperature (from 5 to 80 °C) increased the phthalate migration in PE bags (0.83 to 1.21 μg/kg), especially dibutyl phthalate (80 °C), which was observed at the same temperature (80 °C), DBP migration has been reported to be lower in concentration compared to its similar form, DEHP, in foods. The same study revealed that long-chain DEHP interacts poorly with the polymer and moves easily when heated^32^.

**Figure 6:**
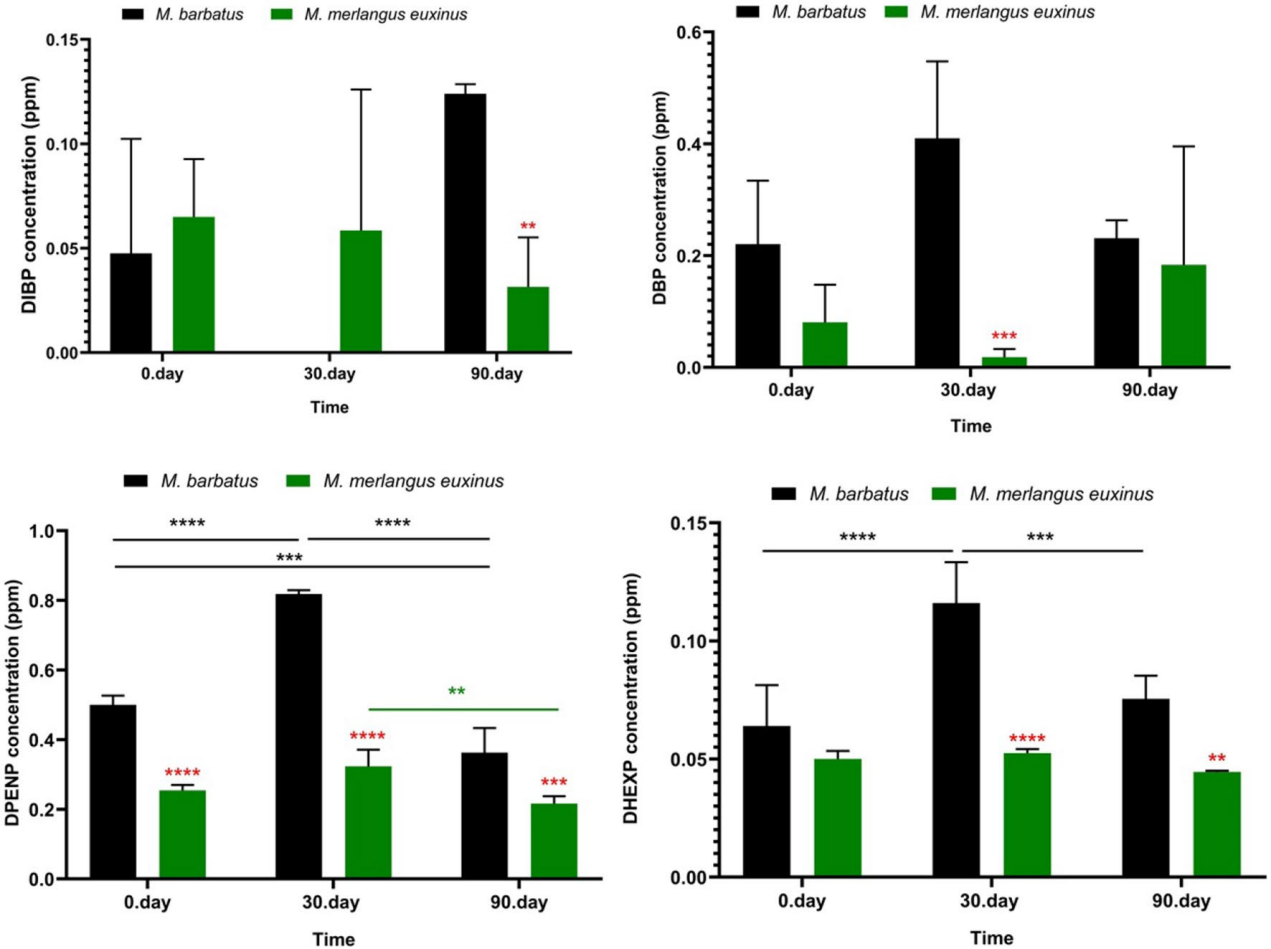
Quantities of different phthalate species in fish tissues: the concentrations (ppm) of DIBP, DBP, DPENP and DHEXP. Each bar represents the mean ± SD of four replicates (n = 4). The degree of significance of the results is represented by following *p* values. (*p < 0.05, **p < 0.01, ***p < 0.001, ****p < 0.0001) (Two-way ANOVA, Bonferroni's multiple comparisons test). Red star: Difference between fish species in the same time groups, green star: Difference between different storage times in *Merlangius merlangus euxinus* species, black star: Difference between different storage times in *Mullus barbatus* species.

**Figure 7:**
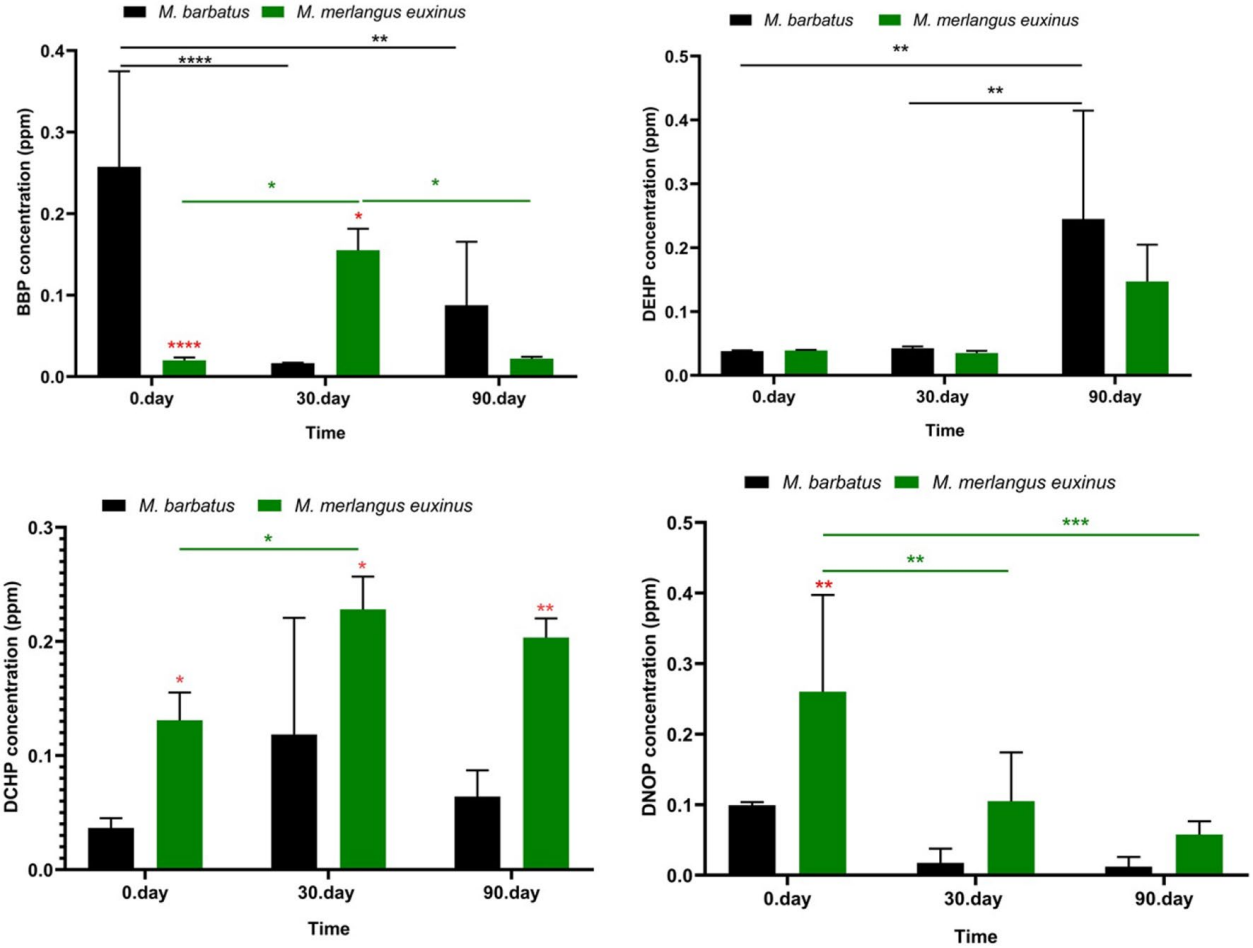
Quantities of different phthalate species in fish tissues: the concentrations (ppm) of BBP, DEHP, DCHP and DNOP. Each bar represents the mean ± SD of four replicates (*n* = 4). The degree of significance of the results is represented by following *p* values. (*p < 0.05, **p < 0.01, ***p < 0.001, ****p < 0.0001) (Two-way ANOVA, Bonferroni's multiple comparisons test). Red star: Difference between the fish species in the same time groups, green star: Difference between different storage times in *Merlangius merlangus euxinus* species, black star: Difference between different storage times in *Mullus barbatus* species.

**Figure 8:**
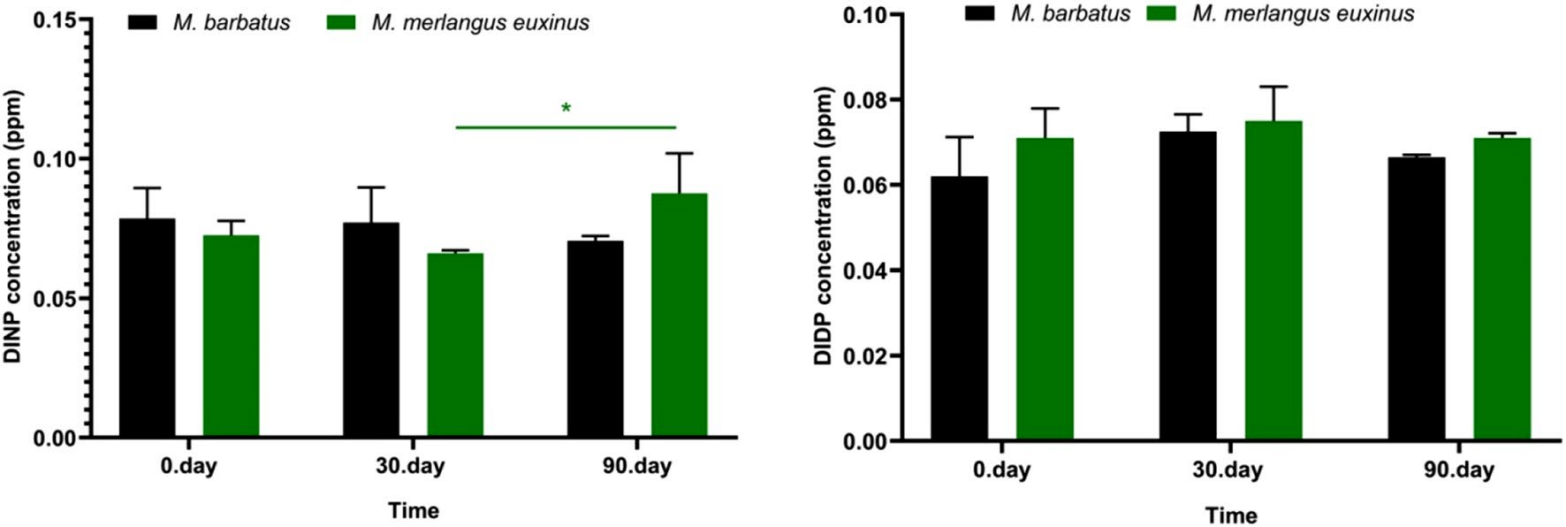
Quantities of different phthalate species in fish tissues: the concentrations (ppm) of DINP and DIDP. Each bar represents the mean ± SD of four replicates (*n* = 4). The degree of significance of the results is represented by following *p* values. (*p < 0.05, **p < 0.01, ***p < 0.001, ****p < 0.0001) (Two-way ANOVA, Bonferroni's multiple comparisons test). Green star: Difference between different storage times in *Merlangius merlangus euxinus* species.

**Figure 9:**
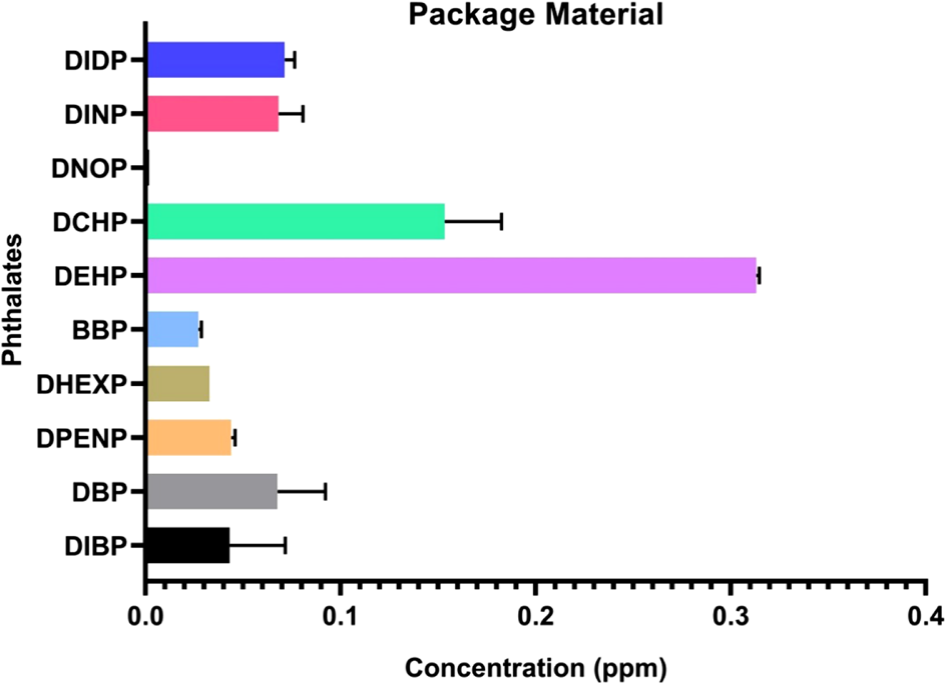
Quantity of phthalates in package material after 90 days of packaging in fish tissues.

In the studies carried out, the presence of microplastics in honey, salt, bottled drinking water, vegetables and fruits has been reported^40–42^. Similarly, there have been some studies reporting the presence of plastic particles (nano, micro, meso, and/or macro plastic) for human consumption in seafood and fish^12,40,43–45^. However, there is no complete study of packaging-mediated phthalate migration and its mechanism yet. This research, designed to fill this gap, is a pilot monitoring study of packaging-mediated migration of PAEs in food and its interaction with storage time (Figs. 6, 7, 8 and 9).

In our research findings, the amounts of PAEs varied according to the fish species. PAEs act as an electron acceptor, and this activity promotes hydrogen bond formation where sorption is accelerated^46^. Migration of plasticizer and the degree of wear of packaging material components may have been effective in the amount of DPENP determined at higher levels than other PAEs homologues in both fish species on the 90^th^ day of storage in our study (Fig. 6–7). This situation for DPENP migration was reported by Velotto^47^ in a study with hot drinks. Our findings showed variations in the amount of PAEs depending on the storage period, which is consistent with the previous studies^48^. It has been reported that increased storage time leads to the formation of higher levels of transported compounds (eg DEHP) in foods^49^.

In our study, PAEs concentrations varied depending on time in PAEs homologues that we found at medium or high levels in the temporal analyzes determined for both fish species (on the 0th, 30th, and 90th days of storage) (Fig. 8). In both fish species, DPENP was the highest concentration of PAEs homologue at all storage times (Fig. 6).

Polymer properties, physical properties and polymer chain dynamics can be affected by different classes of plasticizers. This is important in understanding the migration potential of PAEs in food contact materials (Fig. 9). In particular, it is known that film food packages contain low-density polyethylene polymer type plastics^37^. From this point of view, molecular weight is an important parameter and it is considered as a decision mechanism in the use of phthalates [short alkyl chain DBP, BZBP, DEHP PAEs are used as solvents and adhesives, while high molecular weight (HMW) DINP, DIDP are used as plasticizers]^32^.

Polyethylene (PE) is widely used in reusable bags and food packaging^38^. Polystyrene (PS) is known to be in daily food packaging, disposable plates, cups and materials used in food consumption^39^. In our study, it was determined that the vacuum packaging process and the storage time affect the chemical composition of the package material, and different polymer types were determined in the processed packaged material. Most research supports the concept that high temperature or increased temperature affects the release of microplastics from plastic packaging material^50–52^. In line with previous studies^48^, our research findings showed a positive correlation of PAEs release with storage time. Similarly, it is thought that the storage time is effective in increasing DEHP ester levels in fillets.

Cheshmazar et al.^49^showed that increased storage time was effective in the formation of higher levels of transported compounds (eg DEHP) in foods and reported that factors such as storage time, temperature, sunlight, etc. play a role in the release of PAEs from packaging materials to foods.

## Conclusion

In the packaging material and both fish species, 10 kinds of PAEs homologue (DIBP, DBP, DPENP, DHEXP, BBP, DEHP, DCHP, DnOP, DINP and DIDP) were determined. The presence of PAE's was detected in both fish species during the storage period, albeit in small amounts from the packaging material. It was found that the distribution of different PAEs varied between fish species and that the storage time had a positive effect on the dispersion rates. Although these esters were determined in different amounts, lower values than the individual LOQ were obtained. Analyses of the packaging material have shown that the phthalate esters and quantities contained in the packaging are suitable for food. The obtained information will contribute to the food safety data and fill an important gap in the data libraries created on this subject.However, PAEs in fish products need to be monitored / investigated extensively throughout the food chain and processing procedures. In this sense, it is necessary to create a large data pool for all processed and/or unprocessed, semi-processed products, from raw materials to storage, from packaging material to monitoring other elements in contact with food. In addition, in order to clarify the mechanisms by which PAEs migration is affected, especially food composition and quality, with a multibiomarker approach, different fish species should be followed in controlled environments such as temperatures and storage times.

### Supplementary Information

Supplementary Information.

## Data Availability

The data that support the findings of this study are available from the corresponding author upon reasonable request.
