# Supplementary material for: Evaluation of phthalate migration potential in vacuum-packed
Source: Sci Rep. 2024 Apr 4;14:7944. doi: 10.1038/s41598-024-54730-5 (PMC10995151; doi:10.1038/s41598-024-54730-5)
Supplement: Supplementary file 1 — Supplementary Information. [file 41598_2024_54730_MOESM1_ESM.docx]

**Evaluation of phthalate migration potential in vacuum-packed**

Gonca Alak **^a,*^**, Mine Köktürk **^b^**, Muhammed Atamanalp **^c^**

**^a^** Department of Seafood Processing, Faculty of Fisheries, Atatürk University, 25030 Erzurum, Türkiye.

**^b^** Department of Organic Agriculture Management, Faculty of Applied Sciences, Igdir University, 76000 Igdir, Türkiye

**^c^** Department of Aquaculture, Faculty of Fisheries, Atatürk University, 25030 Erzurum, Türkiye

*Author to whom correspondence should be addressed:

Tel: +90 442 231 2716

E-mail: galak@atauni.edu.tr

**Supplementary information**

**Three Tables**

**Tables caption:**

Table 1. Target/qualifier ions, retention time and linearity range under GC-MS/MS conditions for phthalate analysis.

Table 2. AOAC Manual For The Peer Verified Methods Program Analyte - Recovery Table

Table 3. Validation data: LOD and LOQ, repeatability, measurement uncertainty values

**Ten Figures:**

**Figure caption:**

**Figure 1** The calibration plot belonging to LC-MS/MS system for DIBP.

**Figure 2** The calibration plot belonging to LC-MS/MS system for DBP.

**Figure 3** The calibration plot belonging to LC-MS/MS system for DPENP.

**Figure 4** The calibration plot belonging to LC-MS/MS system for DHEXP.

**Figure 5** The calibration plot belonging to LC-MS/MS system for BBP.

**Figure 6** The calibration plot belonging to LC-MS/MS system for DEHP.

**Figure 7** The calibration plot belonging to LC-MS/MS system for DCHP.

**Figure 8** The calibration plot belonging to LC-MS/MS system for DNOP.

**Figure 9** The calibration plot belonging to LC-MS/MS system for DINP.

**Figure 10** The calibration plot belonging to LC-MS/MS system for DIDP.

**Table 1.**

| PAEs | Target ion (m/z) | Qualifier ion (m/z) | Qualifier ion (m/z) | Retention time (RT) | Linearity range (mg/L) |
| --- | --- | --- | --- | --- | --- |
| DIBP | 223 | 149 | 167 | 4.91 | 0.2-10 |
| DBP | 223 | 149 | 167 | 5.25 | 0.2-10 |
| DPENP | 149 | 219 | 237 | 5.88 | 0.2-10 |
| DHEXP | 149 | 233 | 251 | 6.53 | 0.2-10 |
| BBP | 206 | 91 | 149 | 6.66 | 0.2-10 |
| DEHP | 279 | 149 | 167 | 7.18 | 0.2-10 |
| DCHP | 149 | 167 | 249 | 7.33 | 0.2-10 |
| DNOP | 279 | 149 | 167 | 7.58 | 0.2-10 |
| DINP | 293 | 149 | 167 | 7.80 | 0.2-10 |
| DIDP | 307 | 149 | 167 | 8.51 | 0.2-10 |

**Table 2.**

| Concentration (ppm) | Low (%) | High (%) |
| --- | --- | --- |
| 1000000 | 98 | 102 |
| 100000 | 98 | 102 |
| 10000 | 97 | 103 |
| 1000 | 95 | 105 |
| 100 | 80 | 110 |
| 10 | 80 | 110 |
| 0.1 | 80 | 110 |
| 0.01 | 60 | 115 |

**Table 3**

| PAEs | LOD  µg/kg | LOQ  µg/kg | Reporting Limit (µg/kg) | Recovery (%) | Extended  Uncertainty, % |
| --- | --- | --- | --- | --- | --- |
| DIBP | 0.056 | 0.186 | 0.2 | 90.033 | 3.3 |
| DBP | 0.047 | 0.155 | 0.2 | 89.686 | 2.7 |
| DPENP | 0.044 | 0.145 | 0.2 | 87.100 | 3.8 |
| DHEXP | 0.056 | 0.188 | 0.2 | 92.295 | 6.5 |
| BBP | 0.041 | 0.135 | 0.2 | 90.055 | 4.6 |
| DEHP | 0.293 | 0.976 | 0.2 | 93.031 | 10.2 |
| DCHP | 0.100 | 0.334 | 0.2 | 91.183 | 6.6 |
| DNOP | 0.083 | 0.277 | 0.2 | 85.083 | 5.1 |
| DINP | 0.085 | 0.285 | 0.2 | 94.688 | 10.8 |
| DIDP | 0.109 | 0.364 | 0.2 | 87.300 | 8.8 |


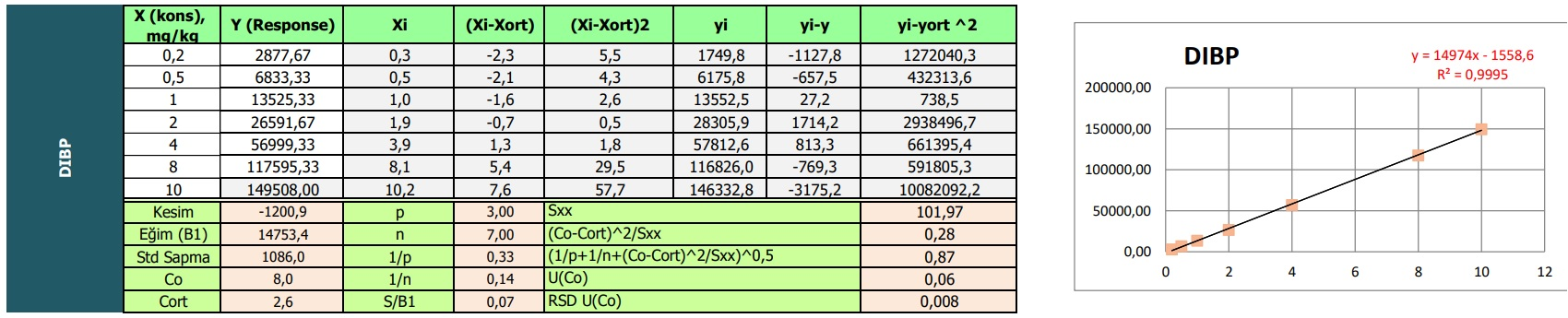


**Figure 1**


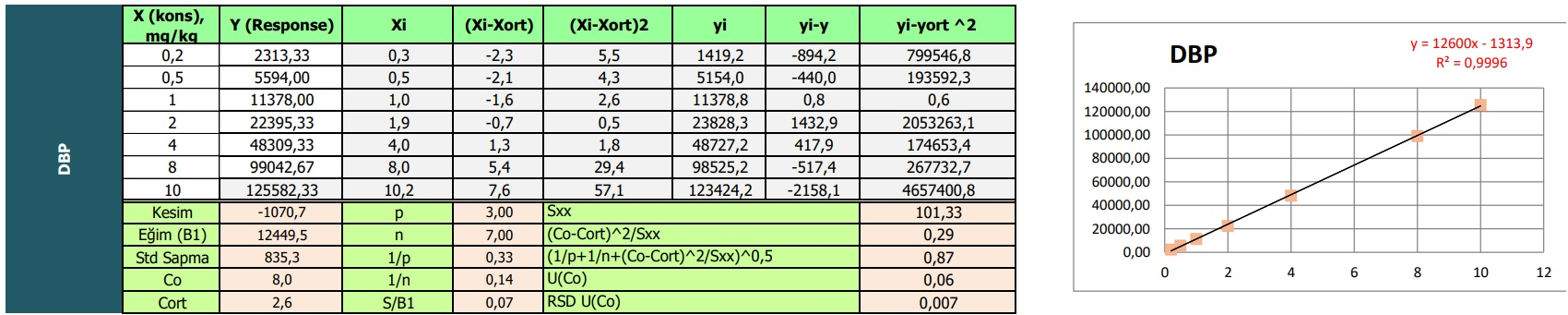


**Figure 2**


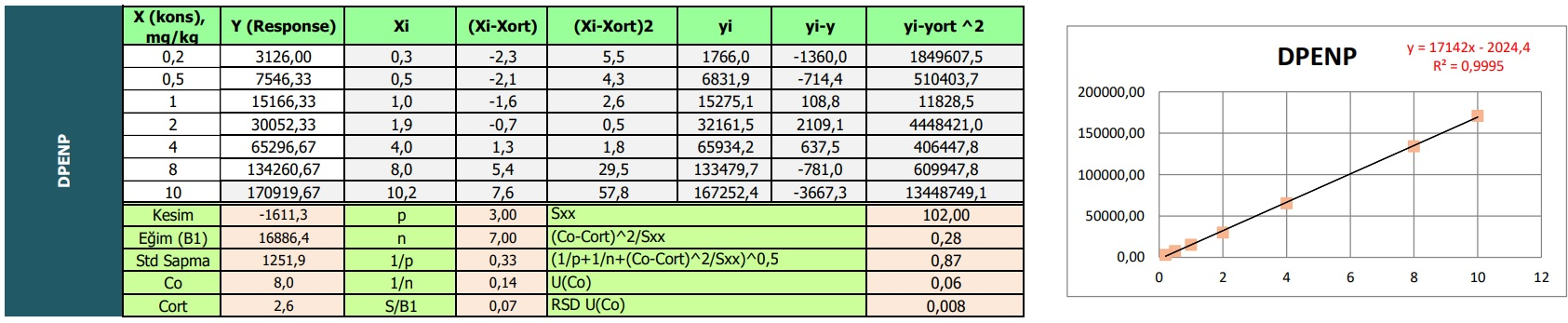


**Figure 3**


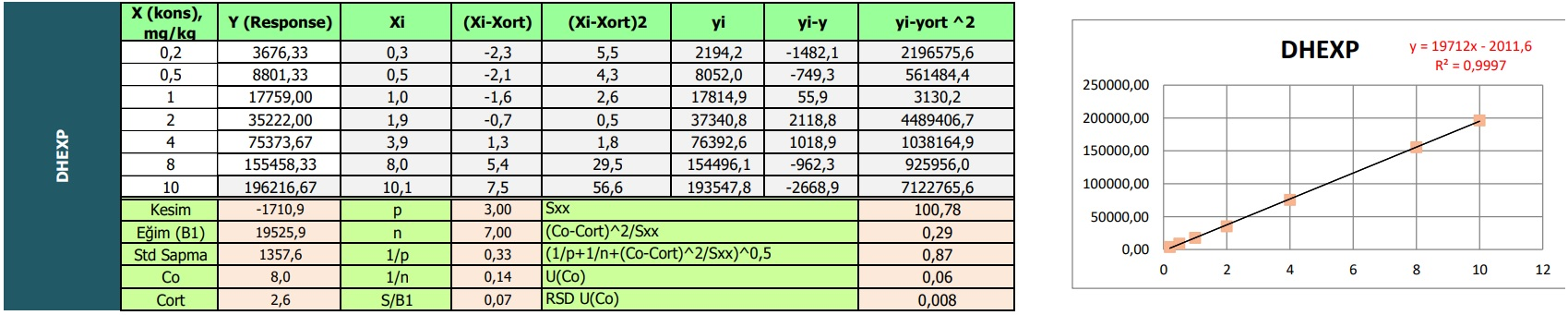


**Figure 4**


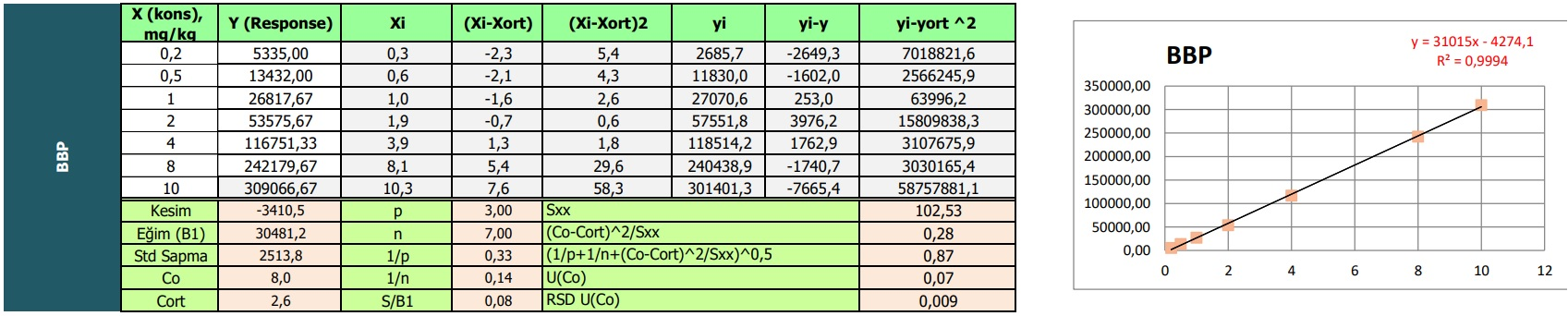


**Figure 5**


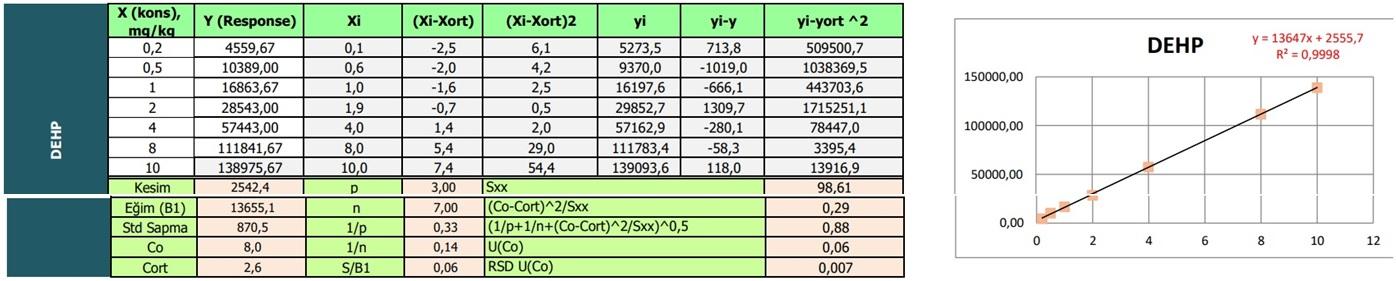


**Figure 6**


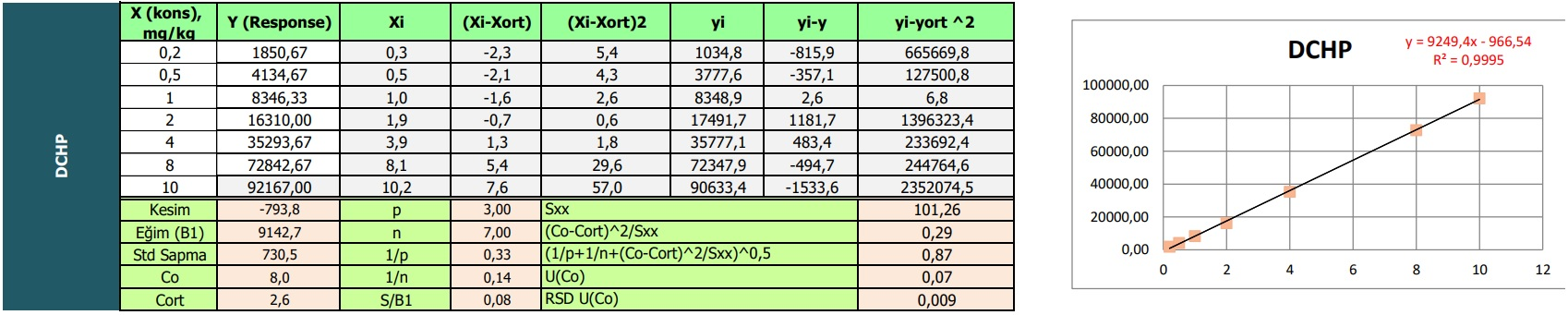


**Figure 7**


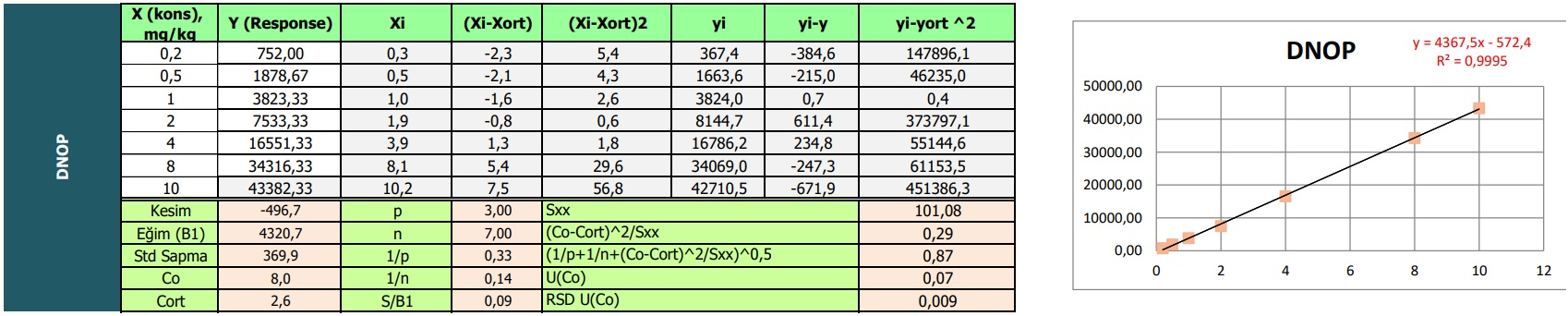


**Figure 8**


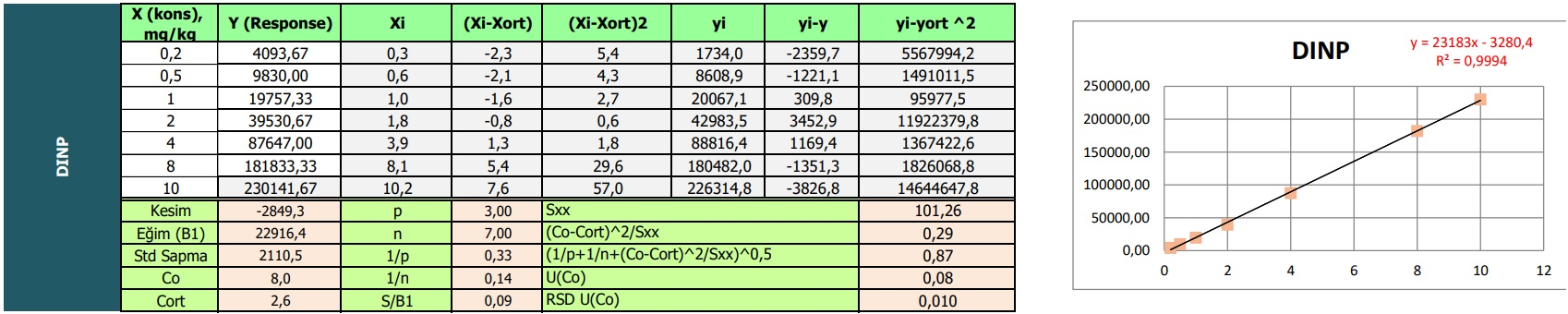


**Figure 9**


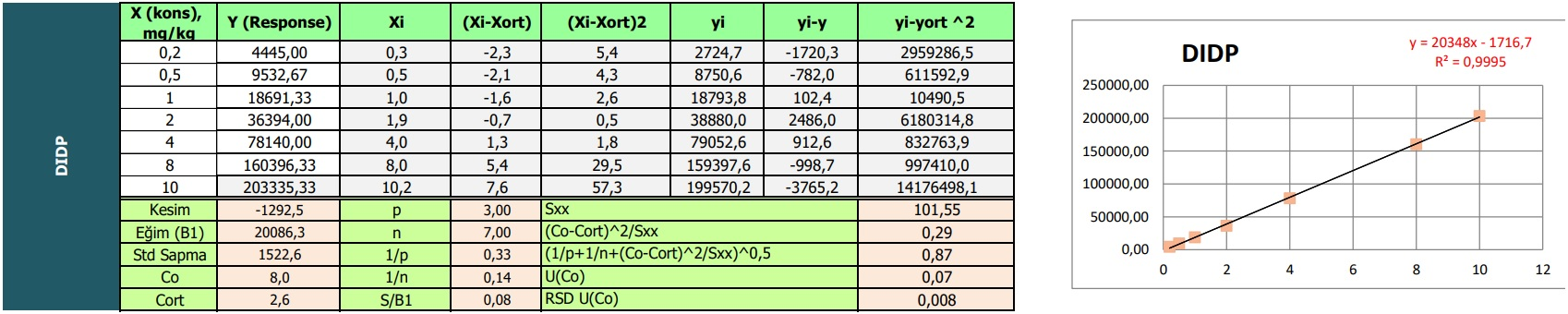


**Figure 10**

**Three Tables:**

**Table caption:**

**Table 1.** Target/qualifier ions, retention time and linearity range under GC-MS/MS conditions for phthalate analysis.

**Table 2.** AOAC Manual For The Peer Verified Methods Program Analyte - Recovery Table

**Table 3.** Validation data: LOD and LOQ, repeatability, measurement uncertainty values

Table1.

| PAEs | Target ion (m/z) | Qualifier ion (m/z) | Qualifier ion (m/z) | Retention time (RT) | Linearity range (mg/L) |
| --- | --- | --- | --- | --- | --- |
| DIBP | 223 | 149 | 167 | 4.91 | 0.2-10 |
| DBP | 223 | 149 | 167 | 5.25 | 0.2-10 |
| DPENP | 149 | 219 | 237 | 5.88 | 0.2-10 |
| DHEXP | 149 | 233 | 251 | 6.53 | 0.2-10 |
| BBP | 206 | 91 | 149 | 6.66 | 0.2-10 |
| DEHP | 279 | 149 | 167 | 7.18 | 0.2-10 |
| DCHP | 149 | 167 | 249 | 7.33 | 0.2-10 |
| DNOP | 279 | 149 | 167 | 7.58 | 0.2-10 |
| DINP | 293 | 149 | 167 | 7.80 | 0.2-10 |
| DIDP | 307 | 149 | 167 | 8.51 | 0.2-10 |

Table2.

| Concentration (ppm) | Low (%) | High (%) |
| --- | --- | --- |
| 1000000 | 98 | 102 |
| 100000 | 98 | 102 |
| 10000 | 97 | 103 |
| 1000 | 95 | 105 |
| 100 | 80 | 110 |
| 10 | 80 | 110 |
| 0.1 | 80 | 110 |
| 0.01 | 60 | 115 |

Table3.

| PAEs | LOD  µg/kg | LOQ  µg/kg | Reporting Limit (µg/kg) | Recovery (%) | Extended  Uncertainty, % |
| --- | --- | --- | --- | --- | --- |
| DIBP | 0.056 | 0.186 | 0.2 | 90.033 | 3.3 |
| DBP | 0.047 | 0.155 | 0.2 | 89.686 | 2.7 |
| DPENP | 0.044 | 0.145 | 0.2 | 87.100 | 3.8 |
| DHEXP | 0.056 | 0.188 | 0.2 | 92.295 | 6.5 |
| BBP | 0.041 | 0.135 | 0.2 | 90.055 | 4.6 |
| DEHP | 0.293 | 0.976 | 0.2 | 93.031 | 10.2 |
| DCHP | 0.100 | 0.334 | 0.2 | 91.183 | 6.6 |
| DNOP | 0.083 | 0.277 | 0.2 | 85.083 | 5.1 |
| DINP | 0.085 | 0.285 | 0.2 | 94.688 | 10.8 |
| DIDP | 0.109 | 0.364 | 0.2 | 87.300 | 8.8 |
